# Improving the Theoretical Understanding Toward Patient-Driven Health Care Innovation Through Online Value Cocreation: Systematic Review

**DOI:** 10.2196/16324

**Published:** 2020-04-24

**Authors:** Atae Rezaei Aghdam, Jason Watson, Cynthia Cliff, Shah Jahan Miah

**Affiliations:** 1 School of Information Systems Science and Engineering Faculty Queensland University of Technology Brisbane Australia; 2 Faculty of Health Queensland University of Technology Brisbane Australia; 3 Business School Victoria University Melbourne Australia

**Keywords:** value cocreation, health care organizations, digital health platforms, online health communities, patient empowerment

## Abstract

**Background:**

Patient participation in the health care domain has surged dramatically through the availability of digital health platforms and online health communities (OHCs). Such patient-driven service innovation has both potential and challenges for health care organizations. Over the last 5 years, articles have surfaced that focus on value cocreation in health care services and the importance of engaging patients and other actors in service delivery. However, a theoretical understanding of how to use OHCs for this purpose is still underdeveloped within the health care service ecosystem.

**Objective:**

This paper aimed to introduce a theoretical discussion for better understanding of the potential of OHCs for health care organizations, in particular, for patient empowerment.

**Methods:**

This literature review study involved a comprehensive search using 12 electronic databases (EMBASE, PsycINFO, Web of Science, Scopus, ScienceDirect, Medical Literature Analysis and Retrieval System Online, PubMed, Elton B Stephens Co [academic], Cumulative Index of Nursing and Allied Health Literature, Accelerated Information Sharing for Law Enforcement, Association for Computing Machinery, and Google Scholar) from 2013 to 2019. A total of 1388 studies were identified from the database search. After removing duplicates and applying inclusion criteria, we thematically analyzed 56 articles using the Braun and Clarke thematic analysis approach.

**Results:**

We identified a list of 5 salient themes: *communication extension, improved health literacy for patients and health care organizations, communication transparency with patients, informational and social support for patients, and patient empowerment in self-management*. The most frequent theme was communication extension, which covers 39% (22/56) of the literature. This theme reported that an extension of communication between patients, caregivers, and physicians and organizations led to new opportunities to create value with minimal time and cost restrictions. Improved health literacy and communication transparency with patients were the second and third most frequent themes, respectively, covering 26% (15/56) and 25% (14/56) of the literature, respectively. The frequency of these themes indicated that the use of OHCs to generate new knowledge from patients’ interactions helped health care organizations to customize treatment plans and establish transparent and effective communication between health care organizations and patients. Furthermore, of the 56 studies, 13 (23%) and 10 (17%) studies contended the opportunity of using OHCs in terms of informational and emotional support and empowering patients in their self-management of diseases.

**Conclusions:**

This review enables better understanding of the current state of the art of the online value cocreation and its potential for health care organizations. This study found that the opportunities for health care organizations through enhancement of patient participation and their cocreation of value in digital health platforms have been rapidly increasing. The identified gaps and opportunities in this study would identify avenues for future directions in modernized and more effective value-oriented health care informatics research.

## Introduction

### Background

The health care industry is under increasing pressure in terms of enhancing their service provision and quality with meeting the growing demands. This is because of population growth and the rise of chronic diseases [1] besides other factors. To respond to these pressures, the health care industry is continuously digitizing its service provisions to provide for more effective and cost-efficient care models, as well as self-care management for personalized health care [2]. As part of the digitization, health care organizations are establishing online health communities (OHCs) as part of their service offering [3] to cocreate value. An OHC refers to a group of people who interact with each other in online environments about similar health issues [4]. This is reflected in the health care literature with growing emphasis on health value cocreation and the benefits of consumer value cocreation in the health domain [5,6]. Historically, value creation was conceptualized as company centric with the value being provided by the company to the customer. More recently, cocreation is viewed as an appropriate customer-centric mechanism for health care organizations in which value is created *with* customers rather than *for* customers [7,8]. In the context of OHCs, the interaction between stakeholders within online platforms can create values that allow stakeholders to share their knowledge and experiences [9]. Through creating value by working with health care organizations, a patient can likely raise their feelings about the existential quality of life, improve the attainment of life goals, and support and reduce their psychological and physical distress [10]. OHCs can provide peer health knowledge, emotional support, and improve self-care for patients with chronic diseases, especially for lifestyle-related diseases such as cancer, obesity or type 2 diabetes [11]. For patients with chronic diseases, OHCs provide a set of anecdotal information [12,13], which helps patients increase their positive emotional experience and attitude toward chronic diseases, engaging them in the activities of the community [14]. Empowering patients improves their role in cocreating, co-designing, and co-delivering health services [15]. In addition, patient empowerment contributes to enhancing the quality of care and health outcomes [16,17]. For instance, OHC users with chronic diseases become more knowledgeable, feel better socially supported, and have improved behavioral and clinical outcomes compared with nonusers [18].

Over the last 5 years, the number of articles that focus on value cocreation in the health care services has increased, highlighting the significance of the collaboration and cocreation of value within the health care service ecosystem between the patients and health care providers [19,20]. In our last paper, we systematically reviewed the literature regarding the role of OHCs as facilitators of value cocreation in the health care service ecosystem within the last 5 years [21]. The findings showed that OHCs provide opportunities for members to cocreate value ubiquitously along with providing members with online informational and emotional support. However, the ability of health care organizations to engage patients in the health care service coproduction and value cocreation has been largely overlooked [22-24]. Due to the importance and apparent oversight of value cocreation for health care organizations, this paper sought to address the following research question: To what extent could online value cocreation add value for health care organizations?

To produce new insights into this research question, this study performed a descriptive literature review to investigate the potential of online value cocreation for health care organizations, identifying the current state of knowledge and the opportunities for health care organizations to engage in online value cocreation.

### Value Cocreation in Health Care

Technological developments promoted a shift from a health care model dominated by professionals toward a patient-centered model in which patients and professionals collaborate to create a service that offers the most optimal health care solution [25]. In recent years, the health care domain has undergone a number of transformations because of the recent advances in technology [26]. In addition, significant priorities for service marketing research include the role of consumers in the cocreation of value within the service sector, the transformative potential of services, and the interface between consumer communities and organizations [27]. This is also reflected in health research with some research evidencing the need for health care service providers and physicians to understand the patient and their role in the provision of health information [26,28,29]. The positive impact of collaborations among patients, physicians, and other actors on health outcomes has justified the significance given to the health care domain in investigating the cocreation of value [30]. Value cocreation in health care is a framework that integrates quality enhancement efforts by health care community staff members with patients’ engagement to promote innovation in creating value [31]. Value cocreation refers to the process through which health care providers collaboratively engage with customers to create value [32]. Organizations are increasingly offering value cocreation opportunities to create more value for both customers and themselves [32-34]. Hence, the provider should take a holistic view of service delivery and consider the important factors in the clinical encounter for empowering patients to assume an active participatory role. Information technology can nurture better health care along with cost reduction and develop service innovation [35,36]. Recently, online community research has gradually started to focus on value cocreation and community outcomes [37]. OHCs provide opportunities for stakeholders such as patients, physicians, caregivers, and health care organizations to access and share health information as well as contribute to the value cocreation process, diminish geographical barriers, and provide informational, social, and emotional support [38,39].

### Online Health Communities as Platforms for Value Cocreation

With the rapid growth of social media technology, OHCs provide opportunities for fostering cocreation among the different stakeholders in health care [10,40]. OHCs are a particular form of special interest community, centered on a shared interest in health conditions and diseases [41]. The primary objective of the most OHCs is to provide a platform for patients regarding interacting with each other to obtain emotional support and disease management and care [42]. Participation in OHCs leads to additional activities carried out by patients, which add value to the patient-provider interactions [43]. Research showed that the more effort patients put into value cocreation activities, the more likely they are to continue with the health care provider, to return to the health care provider when they need treatment in the future, and to recommend the provider to others [44]. In the context of service-dominant logic, which is focused on patients’ contribution to the value creation, the customer is *always a cocreator of value* [8]. In essence, OHCs are changing the way patients treat and manage their health [45]. These communities facilitate self-management through health information exchange and disease experiences [46]. The prominent characteristics of online communities are strong social relationship among members, community-specific organizational structure and way of discussion, history sharing, community rituals, and common online meeting space [47]. All these characteristics support identity for the community, provide a long-lasting relationship between participants, and foster strong member commitment to community purposes [45]. Several studies have focused on the benefits of OHCs, including (a) availability of health-related information especially for people who live in remote areas, facilitating information and social support without the need for driving long distance for face-to-face support group [12,13]; (b) access to health-related information with minimum cost [14]; (c) accessibility to experience-based health information, sharing daily coping habits and user experiences with symptoms [23,48]; and (d) decrease in the feeling of loneliness, creating social interactions among patients with a stigmatized medical condition and avoiding asking for help outside of the online community [23,49]. By empowering patients in OHCs, it is possible to activate value cocreation path between them and health care organizations [50]. As such, by enabling patients to support each other in OHCs, organizations can also indirectly improve customers’ ties with the product and with the organization [46]. Accordingly, OHCs offer cocreation of value opportunities among patients, physicians, and health care organizations to improve health care outcomes [51]. This process, in turn, fosters patients’ access to health information [52].

### Health Care Organizations and Online Value Cocreation

To date, many health care organizations are rapidly recognizing the importance of OHCs as a significant platform of complementary service to improve the total quality of health care services [53]. Although the advantages of using OHCs for health care providers are promising, health care organizations frequently discounted OHCs’ information because of the lack of clinical training of contributors and perceived lower quality of online health information [54]. Health care organizations also experience barriers regarding privacy, confidentiality, reputation management, and the dissemination of inaccurate health information [55-57]. Many health care providers worry about broadcasting misinformation and its negative influence on patients’ health decisions [54,58,59]. Some patients contended that the risk of misinformation in OHCs might be reduced by the participation of health care organization in the conversation. In return, health care organizations could receive valuable experience-based health information from patients, saving organizational resources [32]. The diverse needs of various patients prevent the setup of a single, one-size-fits-all community; rather, cognitive- and affective-related values in a community depend on who participate in the community, the foundation of their relationship, and their activities such as sharing experiences, assessing new ideas, and recommending alternative treatments. Therefore, the complexity of digital services, which involve different goals of interaction among different actors, demands a more granular view of value cocreation in online communities [60]. Although numerous businesses have started to harness the advance potential of online communities by utilizing them as an online environment for customer co-innovation and value cocreation, health care organizations are lagging behind [29,61]. Hence, health care organizations can extend a better understanding of various types of consumer value cocreation that is enabled by OHCs [10]. To address these gaps and to deeply understand the potential of online value cocreation for health care organizations, our main objective was as follows: to identify the salient themes of the current literature regarding the potential of online value cocreation for health care organizations.

## Methods

### Review Protocol

To identify the potential of online value cocreation for health care organizations, we needed to investigate the current literature. For doing so, a descriptive literature review is one of the suitable methods for providing a broad and comprehensive background about the current state of the art [62]. We employed Preferred Reporting Items for Systematic Reviews and Meta-Analyses (PRISMA) checklist to guide our systematic review of relevant peer-reviewed literature [63]. The main aim of the PRISMA is to assist authors in improving the reporting of systematic review and meta-analysis [63]. The following sections explain the article selection process and review protocol.

### Search Method and Article Selection

The purpose of this study was to critically appraise extant work to answer the following question: to what extent could online value cocreation add value for health care organizations? To retrieve relevant articles, we reviewed several academic databases and derived database search terms. The search terms were derived based on keywords in the research question (online value cocreation and health care organizations). However, we did not merely focus on these particular keywords because some related papers discussed the same concepts using different terms. For instance, some articles discussed value cocreation activities in internet-based forums by various stakeholders without using specific terms such as *online value cocreation* or *health care ecosystem*. Hence, to ensure that relevant papers are not neglected, we broadened the search keywords and their synonyms to gather a comprehensive pool of related papers for this study (see Multimedia Appendix 1). Based on our research aim, a search string was defined using Boolean operators such as “AND” and “OR”: (“online value co-creation” OR “value co-creation”) AND (“healthcare organisation” OR “healthcare service providers”) AND (“healthcare service ecosystem”). The variants of the search terms in Multimedia Appendix 1 were applied in different databases. In addition, we considered social science databases such as PsycINFO and EMBASE in terms of covering special studies in psychology and behavioral science. We also looked at controlled vocabularies such as Medical Subject Headings and thesaurus for a more complete search. In addition, three prominent researchers in the field of information systems (IS) and health informatics were asked to recommend any additional studies that met the inclusion criteria.

### Eligibility Criteria

**Figure 1:**
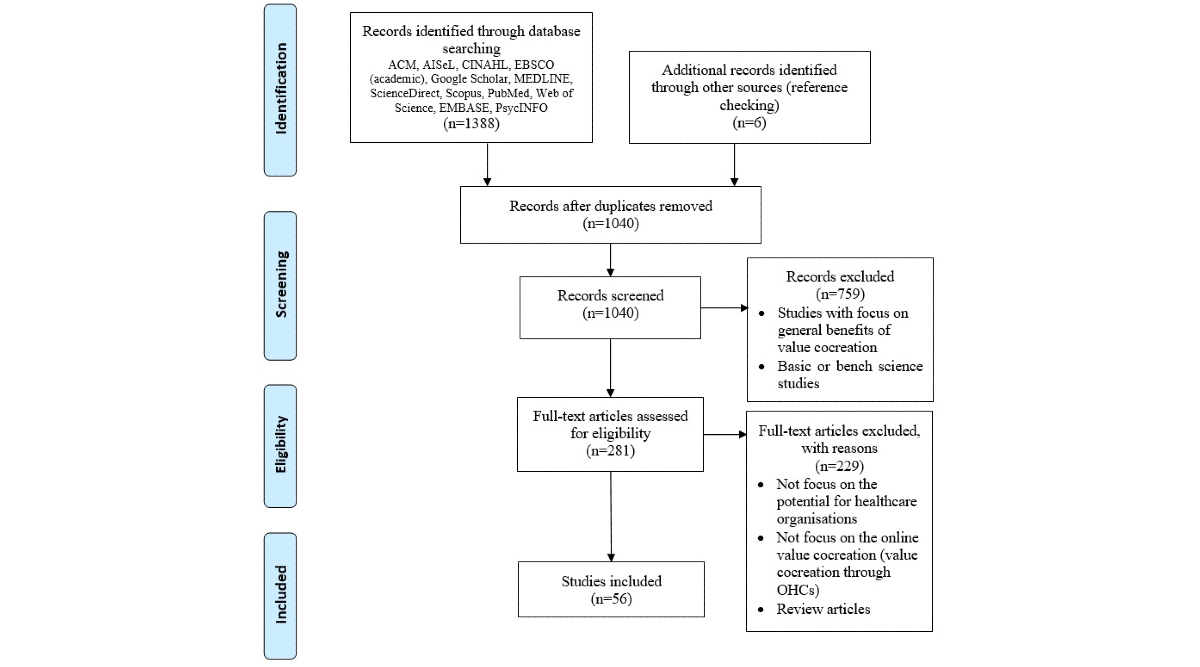
Flow diagram of the literature search. ACM: Association for Computing Machinery; AISeL: Accelerated Information Sharing for Law Enforcement; CINAHL: Cumulative Index of Nursing and Allied Health Literature; EBSCO: Elton B Stephens Co; MEDLINE: Medical Literature Analysis and Retrieval System Online; OHC: online health community.

The searching process was complemented with articles identified from the reference lists as well as searching within the table of contents of selected journals. The outcome of phase 1 yielded 1040 papers after the removal of duplicates. Two scholars separately reviewed the abstracts of each paper and determined if the paper was relevant using the following inclusion criteria: (1) articles were written in English, (2) articles were published between 2013 and 2019, (3) articles focused on the role of health care organizations in the online value cocreation, and (4) articles employed quantitative or qualitative research studies that focused on the use of digital health platforms in value cocreation. During this process, the reference lists of the papers were also checked to identify other articles that are potentially eligible for inclusion. This returned 6 new papers. The two reviewers agreed with each other on the final pool of articles: 281 were identified to be eligible for inclusion in this review. Upon applying the inclusion criteria to the full-text papers, 56 articles were determined to be relevant. Figure 1 (adapted from Nili et al [63]) illustrates the article selection process, and Multimedia Appendix 1 denotes the number of relevant articles retrieved per database. In addition, a summary of the characteristics of the relevant papers is detailed in Multimedia Appendix 2.

### Data Analysis Approach

**Figure 2:**
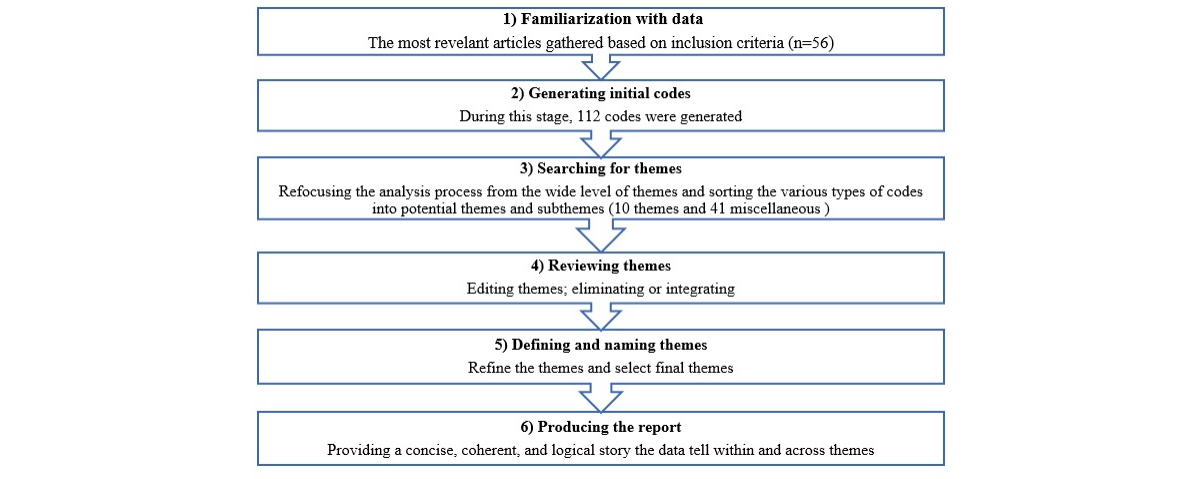
Thematic analysis steps adapted from Braun and Clarke.

To answer the research question, we conducted a thematic analysis following the 6 steps of coding proposed by Braun and Clarke [64] to explore the major themes regarding the potential of online value cocreation for health care organizations. Thematic analysis is a technique that is commonly used to identify, analyze, and report patterns (themes) within data [64]. This technique is an inductive approach and involves coding all sections of findings, discussion, and conclusion of all selected papers (N=56). Applying this method needs *careful reading and rereading of the data* [64] to identify the explicit and implicit meaning embedded within the text [65]. The 6 steps of thematic analysis process defined by Braun and Clarke [64] are collecting data, generating initial codes, searching for themes, reviewing themes, defining and naming themes, and writing the report (Figure 2). We used NVivo 12 (a qualitative data analysis software; QSR International, Melbourne, Australia) as a repository for storing the articles, and all coding was manually performed.

During the first step, we performed a preliminary analysis of the relevant articles and recorded our notes through *memo* and *annotation* features of NVivo and marked ideas for coding in the next step. In the second step, post familiarization with the data, we manually generated an initial list of codes. In total, 112 initial codes were generated. In the third step, after generating initial codes, we refocused the analysis process on the broader level of themes, rather than codes, sorting the different codes into potential themes and subthemes. This was facilitated by creating a thematic map by utilizing the *mind map* features of NVivo to visualize codes and themes. The thematic map revealed 10 themes and 41 miscellaneous codes. In the fourth step, we reviewed and refined themes. In the reviewing process, we reviewed all themes to make sure they followed a coherent pattern. During this phase, two themes collapsed into each other because of their common content. In the refining process, we recoded some additional data that have been missed in earlier stages, resulting in 11 themes. In the fifth step, defining and refining needs to be applied to our themes. In doing so, we named and defined themes [66]. We also performed a detailed analysis of each theme to ensure that the theme was relevant to the research question and that only minimal overlap existed between themes. Another significant factor in this stage is the naming of themes. Names of themes should be concise and punchy and should directly define what the theme is about [66]. In doing so, we named the themes that reflect the answer to our research question. The results of this stage revealed 5 themes (see Table 1). In the sixth step of the thematic analysis, we provided a concise, coherent, and logical report to summarize the themes as presented in the following sections.

**Table 1:** Summary of the thematic analysis outcomes.

Theme	Description	Subthemes	Coverage of the final selection (N=56), n (%)
Communication extension	Digital health platforms such as OHCs^a^ extend communication from the traditional power balance face-to-face consultation between patients and health care professionals to online interactions for facilitating dialogues between stakeholders.	Resource integration Easy access to health care information Resource exchange with other stakeholders	22 (39)
Improved health literacy for patients and health care organizations	The use of digital platforms such as OHCs to generate new knowledge from patients’ interactions and help health care organizations to customize treatment plans, offering some online advice especially for patients with a chronic disease.	Easy-to-read and easy-to-understand health materials Helping patients in the decision-making process Co-learning	15 (26)
Communication transparency with patients	The use of digital platforms such as OHCs establishes transparent and effective communication between patient and patient, patient and physicians, and patient and health care organizations.	Bridge builder Facilitate communications Effective interactions between patients and health care organizations Improve mutual trust	14 (25)
Informational and social support for patients	The use of digital platforms such as OHCs by health care organizations provides informational and social support, which increases the quality of services and patient satisfaction.	Positive comments to patients by health care organizations Timely and appropriate responses to patients Knowledge sharing and information exchange	13 (23)
Patient empowerment in self-management	The use of digital platforms such as OHCs to engage patients in the value cocreation process, assisting them in their self-management of diseases.	Engaging members in OHCs Encouraging members of OHCs Positive patient-provider interactions Self-management intervention for diseases	10 (17)

^a^OHCs: online health communities.

### Trustworthiness

Trustworthiness is a key factor in a qualitative content analysis because text can have multiple meanings and diverse interpretation [67]. It is contended that if the analysis process provides adequate details, the validity of the research will be assured [68]. Accordingly, this research explicates the process of coding step by step to establish the trustworthiness of the study. In terms of testing the trustworthiness of the findings, we employed percent agreement as our method of intercoder reliability checking [69]. Percent agreement is a useful method of checking the reliability of the qualitative findings of less sensitive (eg, literature review) IS projects [69], where the original analyst and a second person compare the findings of their analyses for a sample of papers. The two scholars, experienced in qualitative research and thematic analysis, who checked different parts (paper selection and inclusion and exclusion criteria) placed particular emphasis on reviewing all codes, categories, and themes. They looked at the themes of the study and the way they have originated from categories and codes. During the first meeting, the percent agreement was 75%, and after the second meeting, that is, discussion on the essence of the themes, the general consensus was achieved and the overall results were 100%, making us confident about the reliability of our literature review.

## Results

### Themes

Through performing the thematic analysis, we identified 5 main themes: *communication extension*, *improve health literacy*, *communication transparency with patients*, *patient empowerment in self-management*, and *informational and social support for the patient*. Each theme consisted of several subthemes which are presented in Table 1. The following sections provide more detail into each of them.

As evidenced in Table 1, communication extension was the most explored theme (22/56, 39%) followed by improved health literacy (15/56, 26%), communication transparency with patients (14/56, 25%), informational and social support for patients (13/56, 23%), and patient empowerment in self-management (10/56, 17%).

### Themes Explanation

#### Communication Extension

OHCs provide an opportunity for stakeholders to extend their communication from traditional approach, which comprises few face-to-face scheduled consultations, to effective interactions among the patient, health care professionals, and health care organizations within digital health platforms. In fact, OHCs have a potential to connect members who would never have met each other because of geographical distance. Communication extension assists health care organizations to empower patients to interact with health care professionals and organizations [66]. In this regard, OHCs can contribute to unleashing the provision of health care services and facilitate resource exchange and peer-to-peer social support. In essence, communication extension brings the patients into focus, and OHCs offer a mechanism for value cocreation among stakeholders with minimal time and cost restriction [17,19,46,66-75]. The vast majority of the literature emphasizes information accessing and creating, sharing, and recombination of resources [43,76]. OHCs assist patients to produce health information by sharing their health experiences and cocreate a value within these platforms [77]. Resource exchanges are the mutual actions taken by stakeholders in the health care service ecosystem to access, monitor, share, and integrate resources [75,78,79]. In fact, the communication extension provides health care organizations with the opportunity to look beyond the patient portal and consider how technology nurtures consumers’ relationship, engagement, and contributions as well as the health care organizations to better understand the value cocreation process that occurs within digital health platforms [66,71].

#### Improved Health Literacy of Patients and Health Care Organizations

As indicated in the previous theme, health care organizations need to empower and engage patients in the value cocreation process. However, insufficient organizational health literacy impoverishes the ability of a health care organization to fulfill this task [80]. Health care organizations need to educate patients on their innovation that they are planning to offer to patients, and patients need to educate health care organizations in the context of their everyday use of innovation [71]. OHCs can be an effective tool for knowledge sharing and peer education, and it has the potential to impact patients’ health literacy [46,71]. The new knowledge generated from those patient interactions in OHCs assists health care organizations to customize treatment plans, offering some online treatment options especially for patients with a chronic disease [71]. Some papers contend that by enhancing health literacy, it would be possible to decrease the use of medical services, improving value cocreation with stakeholders and, ultimately, improving the efficiency of the health care system [70,72,81,82]. One of the most relevant papers in this review contends that *knowledge should flow both ways*. It means that patients should be educated by health care organizations and vice versa [71]. In fact, interactions between patients and health care organizations in digital health platforms enable organizations to collect patients’ health-related experiences and information. Therefore, health care organizations benefit from digital health platforms such as OHCs that improve the patient’s ability to use digital platforms to find relevant health-related information and to apply the gained knowledge to address a health issue, which is known as *eHealth literacy* in the literature.

#### Communication Transparency With Patients

Communication transparency between patients and health care organizations is a viable method to provide health care organizations, health care professionals, and patients with new opportunities to cocreate value [83,84]. In this regard, OHCs can provide a proper, transparent, and effective communication between stakeholders [82]. The better health care organizations can effectively interact with patients in OHCs, the more online value will be created. Transparent communication and interaction between health care organizations and patients within OHCs are the fundamental building blocks for online value cocreation. One of the most important elements of having transparent and effective communication is trust. Trust can serve as a significant mechanism in reducing the uncertainty and complexity of exchange and enhancing the credibility of online health information [70]. The level of trust affects patients’ behavior with health care organizations. The transparency of communication is critical for a successful and sustainable response to patients’ health problems. This feature enables both health care organizations and patients to interact directly, allowing them to be actively involved with the value cocreation process [76].

#### Informational and Social Support for Patients

OHCs provide opportunities for stakeholders such as patients, physicians, caregivers, and health care organizations to access and share health information as well as contribute to the value cocreation process, diminish geographical barriers, and provide informational, social, and emotional support [38,39]. Value cocreation process occurs when organizations, stakeholders, and users integrate and renew each other’s resources [85]. In this context, OHCs have immense potential to facilitate the process of value cocreation among actors in the health care ecosystem as well as provide an additional mechanism for obtaining informational and emotional support. Due to the nature of OHCs that provides access to information and coordinated social interaction, they constitute an alternative solution for patients’ needs, such that they likely improve the well-being of individuals and society as a whole [86]. In fact, OHCs have developed as a part of the supplementary service of many health care organizations, where patients are directed to the health care organization’s OHC to receive socioemotional support. OHCs are the major source of informational, social, and emotional support for people with health problems, and members of such communities interact with other stakeholders to seek, receive, and provide different types of supports (informational, social, and emotional) [4,70,82]. Users of OHCs participate and experience the various types of value cocreation via social support exchange within these types of communities [87]. Health care organizations can motivate patients in OHCs’ activities by providing positive feedback to patients who are using digital health platforms. These types of feedback increase the patient’s motivation and serve as a signal that the health care organization approves and welcomes patients’ activities in the health care domain [88].

#### Patient Empowerment in Self-Management

OHCs empower patients in the self-management process through the exchange of health-related information and patient experiences. In other words, OHCs can be used to actively engage and empower patients in their health care journey. Engagement in health care service innovation enables health care organizations to be more proactive, establishing and supporting effective approaches for online value cocreation. The empowerment of patients leads to improving the patients’ role in value cocreation [80]. The patient’s empowerment and engagement contribute to enhancing the quality of health care outcome, and health care organizations have started to recognize the significance of patient empowerment as a driver of patient-centered care. Self-management is where health care organizations can enhance patient participation, supporting patients in controlling their lives [82]. Helping patients with chronic diseases such as type 2 diabetes or asthma to self-manage their condition leads to enhanced quality of care and decreases cost and inappropriate use of health care resources [82].

## Discussion

### Principal Findings

The goal of this study was to improve the theoretical understanding toward patient-driven health care innovation and in this case, identifying the potential of online value cocreation for health care organizations. We conducted a descriptive review of the published papers on the potential of online value cocreation for health care organizations. After analyzing a large number of studies, we now, understand the significance of the digital health platforms in the value cocreation process for both health care organizations and patients by identifying the salient themes of the current literature. These themes were *communication extension*, *improved health literacy for patients and health care organizations*, *communication transparency with patients*, *informational and social support for patients*, and *patient empowerment in self-management*.

Numerous public and nonprofit health care organizations have started to embrace OHCs to support patients’ requirements to locate others with similar health issues and share experiences at a peer-to-peer level [89]. Accordingly, we believe health care organizations perceive OHCs as a tool for extending the communication from the traditional power balanced between health care professionals and patients to online interaction among all stakeholders within the health care service ecosystem, empowering patients in their self-management of diseases by cocreating social and emotional value within OHCs, informationally and socially supporting patients, and establishing transparent communication with patients. Health care organizations might benefit from the interaction between members in OHCs. For example, by monitoring the user-generated content within the community, organizations can gain a deeper and better understanding of members’ needs and finally co-innovate and coproduce health services with customers. This generated knowledge enhances members’ and organizations’ health literacy, helping patients in the decision-making process regarding health care services. In essence, productive collaboration with multiple stakeholders improves the resource, competencies, and capabilities, which fosters values in the cocreation process [85,90]. On the basis of our findings and in line with recommendations to the industry, we propose that health care organizations can prioritize the findings of this study (themes). By prioritizing the salient themes, health care organizations can leverage the potential of online value cocreation to improve their service quality and patient empowerment. According to the recent studies, “the organizations that have been shifting their strategies toward value-based care generally share certain advantages: financial stability, positive relationship with physicians, advanced information systems and (often) affiliation with health plan” [91]. Therefore, this study elucidates the potential that OHCs provide to health care organizations to engage in the value cocreation process. We employed the findings of this review in the real-world program for diabetes group education through conceptualizing OHCs and their potential for this cohort.

### Strengths and Limitations

There are multiple strengths of this systematic review. It was conducted based on the PRISMA guidelines. It employed a rigorous and extensive search strategy to identify the most relevant outlets. Paper selection process based on the inclusion criteria, paper codding, and theme identification were conducted in duplicate by two members of the research team independently to ensure the accuracy of the findings. This study yielded beneficial findings that enabled us to synthesize and present the current state of the art of the potential of online value cocreation for health care organizations. Although this literature review sheds light on the potential of online value cocreation for health care organizations, some limitations of our review need to be considered. Our inclusion criteria limited our review results to only English-language articles and published, peer-reviewed literature between 2013 and 2019. Hence, these restrictions might have led to an exclusion of relevant literature.

### Directions for Future Studies

This review helped us to explore some interesting directions for future work. Future studies can delve into providing a holistic view of the importance of health service coproduction and value cocreation in shaping a dynamic health care ecosystem [92-94]. There is also a lack of understanding on how interactions among stakeholders, especially at the meso and macro levels, contribute to the emergence of value cocreation [95]. Another avenue for future research is to investigate the perspective in which health care organizations are able to engage indirectly in online value cocreation. In fact, the *cocreation of value through engagement in health care warrants more detailed exploration* and highlights the need for more empirical analysis and data on this significant area in health care services [9]. Only a few studies explored the value cocreation at higher levels of health care service ecosystems such as the meso and macro levels. Accordingly, future research can examine the online value cocreation in the health care service ecosystem in the higher levels of the service ecosystem. Future research should be directed toward improving understanding of the engagement level of health care organizations in value cocreation through OHCs. In particular, enhanced understanding of health care organizations’ participation determinants in online value cocreation process and the factors that underpin this phenomenon is required. This may involve future studies with a long follow-up period. The following recommendations for future work might be useful for both health care researchers and organizations:

1. Investigate how health care organizations indirectly engage with online value cocreation process. Identify their challenges and policies for online activities in the virtual communities.
2. Enhance understanding of the level of engagement of health care organizations in online value cocreation and explore health care organizations’ participation determinants in the online value cocreation process.
3. Develop strategies to boost ongoing engagement of health care consumers. This will empower patients in their self-management of chronic diseases.
4. Increase understanding of how health care organizations encourage health care consumers in the health care service coproduction and co-innovation through OHCs.
5. Implement security and data privacy rules considering health care organizations’ perspectives on OHCs, trust-building measures, and challenges associated with the privacy in OHCs.

### Conclusions

The findings of this study enrich our understanding of online value cocreation and its potential for health care organizations by providing a rich review of the literature in online value cocreation. The health care domain can be conceived as a cocreating service system based on the engagement of health care organizations and patients, caregivers, and health care professionals. In this regard, digital platform is one of the most prevalent sources of interaction and online value cocreation. Accordingly, this study aims to improve theoretical understanding toward a patient-driven innovation, such as OHCs, from the value cocreation lenses. Our findings reveal that to foster the implementation of an effective service ecosystem, health care organizations should be able to empower both patients and health care professionals to allow them to actively participate in value cocreation processes in digital platforms such as OHCs. We contend that the outcomes of our study can provide a bird’s-eye view for health care organizations to leverage OHCs for improving their business intelligence along with patient empowerment. Our findings would be useful for health care organization policymakers on how digital health platforms such as OHCs facilitate the value cocreation for health care organizations. Moreover, the findings of this study would be able to guide health care organizations in choosing and implementing strategies and features in their online communities that lead to positive outcomes. Here, we argue that existing OHCs can assist researchers and health care organizations not only by identifying the benefits and potential but also by facilitating value cocreation in the health care service ecosystem.

## Appendix

Multimedia Appendix 1 The variant of search terms in different databases.

Multimedia Appendix 2 Profile characteristics of selected papers.

## Abbreviations

IS: information systems
OHC: online health community
PRISMA: Preferred Reporting Items for Systematic Reviews and Meta-Analyses
