# Supplementary material for: Improving the Theoretical Understanding Toward Patient-Driven Health Care Innovation Through Online Value Cocreation: Systematic Review
Source: J Med Internet Res. 2020 Apr 24;22(4):e16324. doi: 10.2196/16324 (PMC7210492; doi:10.2196/16324)
Supplement: Multimedia Appendix 1 [file jmir_v22i4e16324_app1.docx]

| **Search terms** | **Databases** | **Initial selection** | **Final selection** |
| --- | --- | --- | --- |
| **Main keyword:** “value co-creation”, “digital health platforms”, “online health communities”, “virtual health communities”, “web-based discussion forums” ,“digital health platforms”, “Internet-based forums”, “health online social networks”, “health organi?ations”, “healthcare organi?ations”, “hospitals, healthcare service providers”, “healthcare service ecosystem”, “online value co-creation” | ACM | 101 | 4 |
|  | AISeL | 484 | 7 |
|  | CINAHL | 10 | 5 |
|  | EBSCO (academic) | 35 | 6 |
|  | Google scholar | 472 | 7 |
|  | Medline | 6 | 4 |
|  | ScienceDirect | 68 | 5 |
|  | Scopus | 43 | 6 |
|  | PubMed | 8 | 6 |
|  | Web of Science | 92 | 3 |
|  | Embase | 59 | 2 |
|  | PsychINFO | 10 | 1 |
| **Total** | | **1388** | **56** |

The variant of search terms in different databases present in the following table.
