# Supplementary material for: Improving the Theoretical Understanding Toward Patient-Driven Health Care Innovation Through Online Value Cocreation: Systematic Review
Source: J Med Internet Res. 2020 Apr 24;22(4):e16324. doi: 10.2196/16324 (PMC7210492; doi:10.2196/16324)
Supplement: Multimedia Appendix 2 [file jmir_v22i4e16324_app2.docx]

Demonstrate profile characteristics of selected papers.

| Profile characteristics | Number of papers |
| --- | --- |
| Publication year | |
| 2013 | 4 |
| 2014 | 8 |
| 2015 | 4 |
| 2016 | 11 |
| 2017 | 2 |
| 2018 | 7 |
| 2019 | 4 |
| Country | |
| United States | 14 |
| Australia | 6 |
| Austria | 1 |
| United Kingdom | 14 |
| Germany | 1 |
| Ireland | 1 |
| Canada | 13 |
| Korea | 1 |
| New Zealand | 2 |
| Netherlands | 3 |
| Types of digital health platforms | |
| PatientsLikeMe | 3 |
| Cancer online health communities | 4 |
| Parkinson’s disease online communities | 3 |
| Study Methods * |  |
| Survey | 9 |
| Observation | 8 |
| Qualitative | 26 |
| Quantitative | 6 |
| Case study/case of online forums | 18 |
| *Some studies used mixed-method | |
